# Development of a novel, theoretically motivated scale to assess cognitive learning styles related to the autism spectrum

**DOI:** 10.1186/s12888-022-04334-y

**Published:** 2022-11-11

**Authors:** Seyed Mohammad Mahdi Moshirian Farahi, Craig Leth-Steensen

**Affiliations:** Department of Psychology, Carleton University, 1125 Colonel By Drive, Ottawa, ON K1S 5B6 Canada

**Keywords:** Autism spectrum, Learning style, Individual differences, Scale development

## Abstract

**Background:**

Although theoretical efforts have been made to address the cognitive learning styles of individuals on the autism spectrum, no instrument to measure such learning styles is currently available. The current study aimed to develop such a scale based on the learning style theory of Qian and Lipkin (Front Hum Neurosci 5:77, 2011).

**Methods:**

Response data from total of 768 undergraduate students was used for this study. This sample was split into two subsamples of *N* = 460 and *N* = 308 for exploratory factor analysis (EFA) and confirmatory factor analysis (CFA), respectively. The correlations between the resulting new subscales and some other potentially related measures were examined.

**Results:**

A three-factor structure with 19 items was obtained measuring need for task clarity/familiarity, susceptibility to cognitive load, and the grasping of conceptual relations.

**Conclusions:**

This newly developed measure can be used to help understand the nature of the individual differences in cognitive processing that are evident across both the autism spectrum as well as the overall population more generally.

## Background

The nature of the individual differences in information processing that are related to autistic trait levels at the subclinical level have begun to be studied extensively by researchers interested in how such differences may be related to the autism spectrum [1–7]. Much of this work has attempted to determine how performance on various types of basic cognitive tasks relates to scores on Baron-Cohen, Wheelwright, Skinner, Martin, and Clubley’s Autism-spectrum Quotient (AQ [8]). In this regard, it is important to note that the components of the AQ were derived by Baron-Cohen and colleagues from considerations of what was known at the time about autistic symptomatology as a whole (i.e., difficulties in social skills, communication, switching of attention, and imaginative processing along with enhanced attention to detail). What is lacking, however, is a self-report scale that attempts to tap into specific aspects of the potential cognitive styles associated with autistic information processing. That is, a scale that arises out of specific theoretical notions concerning such styles. The application of such a scale to autistic-trait-related differences in cognitive performance would then allow for much more concrete conclusions to be made about the nature of the information processing underlying any empirical relationships that are found. Namely, such a scale would allow researchers to go beyond simply concluding that a certain aspect of cognitive performance is related to higher levels of autistic traits in general, but is related to a greater affiliation with a particular cognitive learning style that is more likely to be invoked by those on the spectrum. Note that although the two AQ components involving attention to detail and attention switching do indeed attempt to tap into cognitive styles, the use of these two components alone (or simply the AQ total score itself) as predictors of cognitive performance could be regarding as being quite limiting for the field.

Hence, the current work attempts to address this concern by developing a scale based on the theoretical notion furthered by Qian and Lipkin [9] that one aspect of cognition that might serve to distinguish autism spectrum individuals is a tendency to rely more heavily on a look-up-table (LUT) style of learning as opposed to a more interpolation (INT) style of learning. In general, the LUT style “prefers precise, rigid relationships because it aims to store training data precisely” ([9], p. 4). Such a learning style is more favorable to localized, simpler, and rigid tasks with little regularity and structure for generalization (e.g., memorizing the phone book). The LUT learning style applies narrower tuning functions to the learning of examples. The use of narrower tuning function then results in a processing advantage for lower-dimensional feature spaces which naturally are more context-independent. On the other hand, the INT style makes use of broader tuning functions and prefers larger and higher-dimensional feature spaces. The INT style can more efficiently learn and extract regularities from noisy training data which then supports generalization. Thus, INT style is well-suited for more global, flexible, and context-dependent information processing [9].

In this vein, there already are a number of available scales that can be used to study learning styles. For example, Dunn, Dunn, and Price [10] developed a scale based on Dunn and Dunn’s learning style model to measure learners’ preferences in five categories, including environmental (e.g., well-lit), emotional (e.g., motivation), sociological (e.g., studying alone or with others), physical (e.g., perceptual strengths), and psychological (e.g., left vs right brain). As well, the Grasha-Riechmann-Student Learning Style Scales [11] were developed to assess six learning styles referred to as independent, dependent, avoidant, participant, competitive, and collaborative styles. More recently, a somewhat promising Learning Styles Scale was developed by Abdollahimohammad and Ja’afar [12] that involves five factors that they regarded as measuring perceptive, solitary, analytic, competitive, and imaginative learning styles.

Another well-known scale is the Kolb [13] Learning Style Inventory (LSI) developed in 1976 and revised by Kolb in 1999 (see also a more recent shorter version by Manolis, Burns, Assudani, & Chinta, [14]). This scale was developed in accordance with Kolb’s [13] Experiential Learning Model to measure four learning styles (diverging, assimilating, converging, and accommodating) that can be derived from a combination of preferences for concrete experience (CE), abstract conceptualization (AC), reflective observation (RO), and active experimentation (AE). An alternative learning style scale to Kolb’s LSI is the Honey and Mumford (1992) Learning Style Questionnaire (LSQ) that focuses on more on observable learning behaviours rather then learning motivation and preferences. The LSQ and the LSI both assume two combined dimensions, namely, Activist-Reflector (or AE-RO) and Pragmatist-Theorist (or CE-AC [15]).

Nonetheless, there are a number of issues regarding scales such as the LSI and LSQ, that render them a bit problematic for the purpose of predicting performance on basic cognitive tasks. First, the main purpose for their development has been pedagogical. Namely, a concern with the possibility that individuals learn better in different ways and, hence, that knowledge of such differences would enhance learning by providing the opportunity to match the learning process with individual’s preferred learning style [14]. Second, the overall predictive ability of both the LSI and the LSQ has often been regarded as questionable. For example, in some previous work involving a group of 99 human resource management majors, no correlations between any of the subscales of the LSQ and five different academic success criterion measures were present [16]. Most importantly for present purposes, though, such measures were formulated with respect to the typically developing individuals’/learners’ brain.

In order to further our knowledge of learning styles across the spectrum of autistic behaviors, the use of validated measures that are more appropriate for this specific purpose are needed. Despite significant advances in developing measures of autistic symptomatology in adults associated with instruments such as the AQ [8] (see also Hurley, Losh, Parlier, Reznick, & Piven’s Broader Autism Phenotype Questionnaire [17] and more recently Barrett, Uljarević, Baker, Richdale, Jones, & Leekham’s Adult Repetitive Behavior Questionnaire-2 [18]), there currently are no learning style measures that take a specific theoretical view of autistic cognitive/perceptual processing style into account.

Hence, the aim of present study was to develop a learning styles scale based on the work of Qian and Lipkin [9] that can be used to predict and explain cognitive-based performance both for those on the spectrum as well as for the general population given the oft-presumed continuous nature of the spectrum [19]. In the following sections of this report, with a view to this purpose, a scale was derived and its internal structure tested using both exploratory and confirmatory factor analyses. The relationships between each of the subscales of this newly developed scale and the subscales of both the AQ and the LSQ were then examined in order to determine the degree to which each of the facets being measured by this new scale were related to the specific known facets of those two other scales. In addition, because it was felt that some features of the construct of systemizing might well be related to the Qian and Lipkin [9] learning styles, the relationship between this new scale and the Systemizing Quotient (SQ [20]) was also examined.

## Methods

### Participants

Nine hundred and forty-seven undergraduate students (69.1% female and 28.9% male, age M = 19.95, Min = 17, Max = 70) from Carleton University participated online (using Qualtrics) for course credit. As noted in the Results section, data for *N* = 768 could actually be used. All participants provided their written informed consent on the Qualtrics platform before being able to start the survey. This study was approved by the Carleton University Research Ethics Board-B (#106,314).

### Measures

All measures relevant to this study were included in a larger questionnaire set that was developed for a course in psychometrics at Carleton University. The order of administration of the questionnaires for this study is the same as the order that they are reported in the following paragraphs, except for the fact that the initial learning scale items developed by the first and second authors ended up being responded to before and after the AQ, respectively.

The novel Cognitive Learning Styles (CLS) scale was motivated by the theoretical proposals of Qian and Lipkin [9]. Based on their respective readings of Qian and Lipkin [9], the two authors of this study came up with a pool of 60 items designed to tap into LUT and INT learning styles (the full set of items is listed in Table 1). As a first step, the first author designed an initial set of 32 items. The construct relevancy and clarity of these items were then assessed by both the second author (the psychometrics course’s instructor) and three other Psychology graduate students in the course. A short description of Qian and Lipkin’s learning styles (analogous to that given earlier in the Introduction) was provided to the three graduate students in order to familiarize them with the theoretical aspects of the scale. A content validation questionnaire was used to rate the relevancy and clarity of each item on a three-point scale (Relevancy: “*Not very relevant*”, “*Moderately relevant*”, “*Very relevant*”; Clarity: “*Not very clear*”, “*Moderately clear*”, “*Very clear*”). The raters’ agreement with each item was calculated in terms of a percentage (i.e., rating divided by 3 × 100) where items with a mean agreement percentage lower than 70% were eliminated [21]. On the basis of this criterion, 3 items were dropped from the pool (leaving 29 items). Next, the second author worked up an additional set of 31 items to help ensure the most complete coverage possible of the LUT and INT learning style constructs [22]. This resulted in an initial pool of 60 items to be further analyzed as detailed in the following Statistical Analysis section.

**Table 1:** Four-factor solution derived from the initial EFA

	Factor 1	Factor 2	Factor 3	Factor 4
A1 I tend to solve problems step-by-step	.378	-.279		
**A2 I find it easier to solve problems when I have clear instruction**	**.756**			
**A3 I am better when memorizing things that have clear structure**	**.661**			
A4 I prefer to do tasks that I have done before than tasks that are new to me	.668		-.358	
**A5 I perform better when I am asked to do a task that I already know**	**.799**			
**A6 My performance is poor when I have to do two tasks at the same time**		**.580**		
**A7 I am able to listen and read at the same time**^a^		**-.493**		.207
A8 I prefer tasks that demand different skills such as hearing and reading at the same time		-.489		.379
**A9 I make more mistakes when I have a lot to do**		**.479**		
A10 I am flexible in performing different types of tasks		-.271	.367	
**A11 I prefer tasks that are predictable**	**.646**			
*A12 It is easy for me to apply my current knowledge to other tasks*	*.270*		*.441*	
A13 It is difficult to perform tasks that demand new regularities	.243	.323	-.247	
**A14 I learn content better when it has clear instructions**	**.740**		-.271	
A15 I prefer to multitask		-.463		.308
**A16 I am easily distracted by new stimuli**		**.612**		
A17 I easily adapt given redundant information				
A18 I prefer to focus on details than the whole	.302			
**A19 It is hard for me to memorize details**^a^		**.431**		.210
A20 When there are less options to choose, I make better decisions	.365	.209		
**A21 I get overwhelmed when I have to choose one option among different possible options**	.207	**.516**		
A22 I am better with rigid relationships than with flexible relationships				
A23 I do not go beyond the instructions as I believe that this is the best way to determine the solution	.397		-.336	.280
*A24 I prefer to have precise training than flexible training*	*.454*		*.251*	
A25 I easily find specific words in a paragraph of text		-.248	.242	
A26 I perform poorly when I am asked to figure out the main idea when reading		.246	-.219	.325
A27 More information/data regarding a problem that I am solving is always helpful	.317		.234	
A28 I have a hard time separating the signal from noise		.306		.275
**A29 I get overwhelmed when I have too many tasks to do**		**.653**		
A30 I feel that it would be easy for me to learn a bunch of unrelated things		-.309	.370	.242
A31 I tend to remember more general information about things			.324	
**A32 It’s hard for me to learn things one-by-one**^a^				**.552**
*A33 I’m always looking two steps ahead*			*.393*	
*A34 I like to learn things exactly*	*.439*		*.255*	
A35 I am great at dealing with new situations		-.289	.399	
A36 I prefer to view things in isolation	.273			.301
A37 I hate it when I have to decide between two things that differ on many dimensions	.239	.317	.237	
*A38 I like being precise about things*	*.446*		*.284*	
A39 Things need to be quite different for me to be able to tell them apart			-.284	.463
A40 I tend to take things too literally		.204		.367
*A41 Learning new things makes me forget old ones*		*.395*		*.241*
A42 I never take anything at “face value”			.258	.273
**A43 I realize that everything has nuances**		.270	**.572**	
**A44 Most things tend to have very unique meanings**		.219	**.611**	
**A45 I am good at “filling in the gaps” when I am learning something**			**.677**	
A46 A lot of things are not very complex		-.218	.220	.268
**A47 It is easy for me to learn things that can be grouped together**^a^	.211			**.506**
**A48 My memory for previous things is not affected by learning new ones**^a^		**-.510**	.212	
**A49 I can easily tell things apart**			**.569**	
A50 I do not need to learn things precisely	-.236	-.200		.215
A51 I like it when I have to decide between two things that differ according to a single dimension		-.235		
**A52 I always try to see connections between things**			**.621**	
**A53 I prefer situations that I am used to dealing with**	**.665**			
*A54 I do not like it when something seems fuzzy to me*	*.393*		*.221*	
A55 I never have trouble predicting what is going to happen next			.348	
A56 I am not good at “intuiting” things			-.406	.280
A57 I often fail to see the relation between things			-.468	.418
**A58 I always assume that there is “more to the picture than meets the eye”**		.335	**.675**	
**A59 I tend to remember specific details about things**			**.575**	
**A60 Most things have meaning in relation to other things**		.206	**.697**	

Items that are bolded were retained and only factor loading above ± .200 are reported in the table. *Italic *items are cross-loadings.

^a^ These items were later dropped from the scale on the basis of the CFA results

With respect to the response scale, there has been some controversy over the optimal number of response options to use to respond to items such as these, with at least five scale points being recommended in one fairly recent methodological report [23]. Hence, the decision was made to use a five-point Likert scale (“Strongly disagree”, “Disagree”, “Neither agree nor disagree”, “Agree”, Strongly agree”) to respond to the CLS items. Information regarding the final scale factor structure and reliabilities is provided in the following Results section.

The Autism-Spectrum Quotient (AQ [8]) is composed of 50 items (e.g., “*I enjoy social chit-chat*”, “*When I talk, it isn’t always easy for others to get a word in edgewise*”, “*I notice patterns in things all the time* “, “*I tend to have strong interests which I get upset about if I can’t pursue*”, and “*I find it very easy to play games with children that involve pretending*”) assessing five principal dimensions of autism spectrum conditions (10 items each), namely Social Skills, Communication, Attention to Details, Attention Switching, and Imagination. In the present study, AQ data was analyzed using a scoring system based on a four-point Likert scale (from definitely disagree to definitely agree). The total score is the sum of all items (with 26 of them needing to be reversed scored), ranging from 50 to 200. Higher scores indicate greater levels of autistic traits. The Cronbach alpha internal consistency of the AQ obtained from the sample of *N* = 768 participants of the current study was 0.82. Correspondingly, the internal consistencies of the five AQ subscales were 0.75, 0.66, 0.60, 0.54, and 0.59, respectively, here.

The Systemizing Quotient (SQ [20]) is designed to measure systemizing tendencies involving an interest in understanding how things work or are structured (e.g., “*When I look at a building, I am curious about the precise way it was constructed*” and “*I find it difficult to read and understand maps*”). It is composed of 25 items and is responded to using a four-point Likert scale (i.e., with values of 1 – 4 from strongly disagree to strongly agree). All item scores are summed (after reverse-scoring 13 items) with a minimum total score of 25 and a maximum of 100. The Cronbach alpha internal consistency of the SQ obtained from the sample of *N* = 768 participants of the current study was 0.83.

The Learning Style Questionnaire (LSQ [24]) is an 80-item scale that measures four learning styles (20 items each), including Reflector (e.g., “*It's best to think carefully before taking action*”), Activist (e.g., “*I actively seek out new experiences*”), Pragmatist (e.g., “*I can often see better, more practical ways to get things done*”), and Theorist (e.g., “*I am keen to reach answers *via* a logical approach*”). Typically, the answer to each statement is based on “Agree” or “Disagree” such that agreed items are scored for each learning style. Here, to render the scoring comparable to the CLS scale, the LSQ was responded to here using a five-point Likert scale (from strongly disagree to strongly agree). Each learning style has a minimum possible score of 20 and a maximum possible score of 100. The Cronbach alpha internal consistencies of the four LSQ subscales obtained from a sample of *N* = 768 participants of the current study were 0.81, 0.73, 0.72, and 0.69, respectively.

### Statistical analysis

After dropping participants who did not provide enough data to be properly analyzed (see the next section), any further missing responses to the items in the initial 60-item pool of potential CLS items were imputed using the Expectation–Maximization (EM) procedure in the Missing Value Analysis routine in SPSS. In the EM algorithm, the two steps, Expectation (E) and Maximization (M), are iterated using maximum likelihood (ML) multiple times, according to the number of specified iterations. In the E-step, the distribution for the missing data is calculated based on the known values for the observed data and the estimates of sums and sums of squares and cross products (SSCP) are generated. In the M-step, the unknown values are estimated by using all other variables as predictors in a regression model and the SSCP matrix obtained in the E-step is used to estimate a new covariance matrix and associated regression coefficients. The two-step procedure is repeated for several iterations until the differences between the estimated covariance matrices falls below some specified convergence criterion [25].

To determine the nature of the factor structure underlying the initial pool of 60 CLS items, a set of exploratory factor analyses (EFAs) were performed on the set of responses to the 60 CLS items. Given the ordinal nature of the Likert scale used to respond to each item, categorical factor analyses were conducted in Mplus (with all imputed values rounded to the nearest integer). For these EFA analyses, the robust maximum likelihood (MLR) estimator was used in order to obtain the various information criteria (i.e., AIC, BIC, and the sample-size adjusted SSBIC) to be used to determine the best fitting fit model. Factor models specifying 1 to 10 factors were run and the model with the best fit was determined by examining the BIC [26] with lower values indicating better fits. Before running the EFAs, the Bartlett test of Sphericity and the Kaiser–Meyer–Olkin (KMO) measure were examined in SPSS in order to check whether the correlations between all items differ from zero and the sampling adequacy, respectively [27]. The default in Mplus is to assume correlated factors and provide an oblique GEOMIN rotated factor pattern matrix of loadings. With respect to the items to be retained for each factor, loadings of 0.40 or more were regarded as the retention cut-off, provided that they were also at least twice the value of the next highest loading for that item on any other factor (i.e., no substantial cross-loading [27, 28]).

After deciding upon an internal structure (i.e., the factors and their items) for the CLS scale through EFA, a categorical confirmatory factor analysis (CFA) of that factor structure was performed in Mplus using the robust weighted least squares (WLSMV) estimator in order to obtain chi-square related goodness of fit measures. With respect to those fit measures, RMSEA (≤ 0.08), CFI (≥ 0.90), and TLI (≥ 0.90) were considered adequate fit in the model and RMSEA (≤ 0.06), CFI (≥ 0.95), and TLI (≥ 0.95) were regarded as a good model fit [29]. Note that SRMR is not provided for categorical CFA in Mplus.

Given that males are typically over-represented on the autism spectrum but under-represented in the current sample, the degree to which the current factor results were indeed invariant across females and males could be regarded as important information. Hence, categorical CFA-based invariance models were also run in Mplus [30]. Within such an approach, a series of multigroup models are ran with an increasing level of equality constraints applied. First, the fit of a baseline model with all item thresholds (i.e., which replace the intercepts in categorical CFA) and loadings free to vary across the groups (i.e., males and females) was examined to determine whether the same form of the model is adequate for both groups. For categorical CFA invariance models, it is recommended that the next step be a test for threshold invariance by running the model with all thresholds held constant across groups. The third step was to then test for both threshold and loading invariance. To determine invariance, chi-square-based DIFFTEST results were examined (given the nested nature of the models) with nonsignificant tests indicating invariance. As well, recommended changes in both RMSEA (< 0.010) and CFI (< 0.005) for unequally sized groups were also considered [30].

For each of the subscales corresponding to each of the resulting CLS factors, descriptive summary statistics were reported and floor and ceiling (F/C) effects were tested to identify the presence of response bias for either the lowest (floor) or highest (ceiling) score of the subscale. F/C effects were regarded as occurring if the proportion of either the floor or ceiling responding was ≥ 15% [31]. As well, for each CLS subscale, measures of internal consistency (Cronbach’s alpha) were computed. Finally, correlations of each of those subscales with the five AQ subscales, the SQ total score, and the four LSQ subscales were then also obtained. Given the theoretical underpinnings of this work, subscales related to aspects of LUT- and INT-learning, respectively, were expected to be found. If indeed such learning is a refection of the nature of the processing occurring across the autism spectrum, the presence of some convergent relations between those subscales and the five AQ subscales (and, as mentioned, likely also the SQ) should be observed. On the other hand, given the qualitatively different theoretical approach underlying the development of the present cognitive learning style scale and the other previous learning style scales, any relations between the CLS and LSQ subscales revealed by this work would simply help to situate the new CLS subscales in terms of those previous LSQ subscales.

## Results

The total number of participants who responded to the full questionnaire set was *N* = 947. One hundred and fifty-nine respondents who did not respond to at least half of the CLS items were dropped (with 24 not responding to any items in the survey, a further 40 not responding to any items in the survey past the first scale, a further 61 of these respondents not responding to any of the CLS items, and a final 34 not responding to any of the second set of 31 CLS items). For the remaining *N* = 788, 147 missing item responses were then imputed using the EM procedure and subsequently rounded to the nearest integer. Mahalanobis distances were then computed for each participant’s set of CLS item responses. The 20 respondents with the highest distances were then regarded as being careless responders and were also dropped [32]. The final sample of *N* = 768 respondents (69.9% female and 28.9% male) was then randomly split into two subsamples of *N* = 460 (69.8% female and 27.8% male) and *N* = 308 (70.1% female and 29.2% male) for EFA and CFA purposes, respectively. Note that the splitting of the dataset allowed for a confirmatory cross-validation of the factor structure suggested by the EFA on a separate sample which then serves to minimize any potential overfitting issues regarding that initial EFA [33]. In order to provide as many cases as possible for the invariance analyses, however, these were run on the full final sample of *N* = 768.

With respect to the EFA, the KMO and Bartlett’s test verified the appropriateness of the factor analysis (KMO = 0.823, *p* < 0.001). The EFA model fit results across all of the potential factor solutions are given in Table 2. The lowest value for the BIC was obtained for the five-factor model. However, given that the BIC for the four-factor model was so close to that value as to be essentially indistinguishable, the more parsimonious four-factor model was considered to be the optimal model moving forward. The factor loadings for the four-factor model are listed in Table 1 with all loadings greater than 0.20 reported. The items retained for each factor (according to the criteria outlined previously) are those bolded in Table 1.

**Table 2:** Model fit values from the set of EFAs

Factors	-2LL	AIC	BIC	SSBIC
1	-34,732	70,063	71,298	70,349
2	-33,607	67,930	69,409	68,273
3	-33,305	67,442	69,161	67,840
4	-33,049	67,044	68,998	67,497
5	-32,875	66,810	68,995	67,316
6	-32,726	66,620	69,332	67,179
7	-32,615	66,505	69,141	67,116
8	-32,680	66,742	69,597	67,404
9	-32,450	66,385	69,455	67,096
10	-32,374	66,335	69,615	67,095

**Fig. 1:**
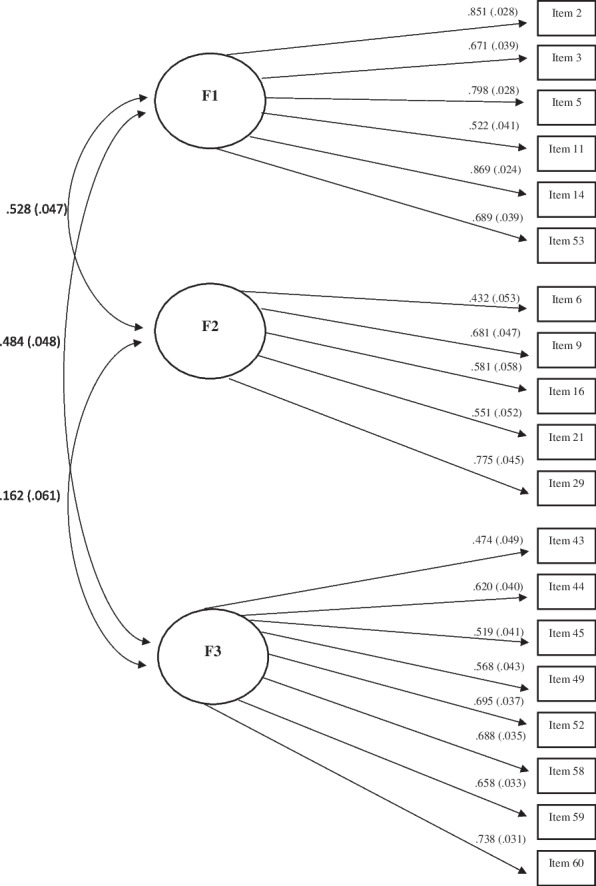
Final Factor Solution

The resulting model fit values for the subsequent CFA on those retained items were RMSEA = 0.070, CFI = 0.903, and TLI = 0.893 which suggests an adequate level of fit according to both the RMSEA and the CFI values. For Factor 1, the resulting loadings for Items 2, 3, 5, 11, 14, and 53 were 0.855, 0.669, 0.790, 0.510, 0.870, and 0.702, respectively. For Factor 2, the resulting loadings for Items 6, 7, 9, 16, 19, 21, 29, and 48 were 0.477, -0.311, 0.647, 0.587, 0.303, 561, 838, and -0.303, respectively. For Factor 3, the resulting loadings for Items 43, 44, 45, 49, 52, 58, 59, and 60 were 0.473, 0.635, 0.530, 0.583, 0.682, 0.679, 0.645, and 0.745, respectively. For Factor 4, the resulting loadings for Items 32 and 47 were -0.334 and 0.615, respectively. After removing the four items with CFA loadings that were less than 0.400 (Item 7, 19, and 48 of Factor 2, and Item 32 of Factor 4) as well as omitting the lone remaining item in Factor 4 (Item 47; which then deleted that factor), the resulting model fit values for the CFA on the final set of 19 retained items were RMSEA = 0.064, CFI = 0.945, and TLI = 0.937. This three-factor model with the loadings and factor correlations is shown in Fig. 1. This model represents the version of the CLS scale that is recommended for use moving forward.

For this final model, the results of the first invariance test regarding the equality of the form of the model indicated an adequate model fit (see Table 3). Equating the factor thresholds did not then result in a significant diminishment of the model fit (see Table 3). Further equating the factor loadings did, however, result in a significant diminishment of the model fit (see Table 3) although the changes in the goodness of fit measures (i.e., ΔRMSEA, ΔCFI) were very small and not indicative of non-invariance. Moreover, further examination of loading invariance for each factor separately indicated that equating the loadings for Factor 1 only resulted in a significant diminishment of fit (see Table 3) although, again, the changes in the goodness of fit measures were not indicative of non-invariance. Loadings for Items 2, 3, 5, 11, 14, and 53 of Factor 1 with those for the other two factors held constant were 1.064, 0.656, 0.940, 0.507, 1.009, and 0.571 for the males but 0.765, 0.707, 0.737, 0.551, 0.844 and 0.717 for the females, respectively.

**Table 3:** Results of the Invariance Tests

	CFI	TLI	RMSEA	χ^2^	df	ΔCFI	ΔRMSEA	LRT	Δdf	*p*
Model invariance	.939	.929	.064	758	298					
Threshold invariance	.939	.938	.060	796	336	0	.004	38	38	.468
Loading invariance	.941	.942	.058	825	352	-.002	.002	29	16	.022
Factor 1 Loading invariance	.938	.938	.060	812	341	.001	0	16	5	.008
Factor 2 Loading invariance	.940	.939	.059	801	340	-.001	.001	5	4	.328
Factor 3 Loading invariance	.941	.941	.059	806	343	-.002	.001	10	7	.183

With respect to the CLS factors, CLS1 (Need for Clarity/Familiarity – 6 items) indexes how important it is to an individual to have clear instructions and problem structure when learning or performing a task along with a preference for doing things that are known to them. CLS2 (Susceptibility to Load – 5 items) indexes the extent to which higher cognitive load has a deleterious effect on an individuals’ cognitive processing. CLS3 (Conceptual Relations – 8 items) indexes a general tendency to be able to conceptualize things both as unique and related entities. Even though the factor analysis results necessarily indicate that each of these CLS factors accounts for unique variation in the final retained set of CLS items, the pattern of factor correlations in Fig. 1 indicates that CLS1 (Need for Clarity/Familiarity) is related positively to both CLS2 (Susceptibility to Load) and CLS3 (Conceptual Relations). On the other hand, the CLS2 and CLS3 factors are related to a lesser extent.

Coefficient alpha reliability values, computed from the full sample of *N* = 768, were 0.804, 0.712, and 0.790 for each of the CLS subscales, respectively. Mean subscale scores for the full sample, that were derived by summing the corresponding item scores, are provided in Table 4 for each of the CLS subscales. Given that the mean score for the CLS1 was approaching the maximum score, the presence of potential ceiling effects was examined. In this vein, the frequency of those scoring with the highest possible value (i.e., 30) of the CLS1 scale was 55 or 7.1% which is lower than the criterion of 15% (although note that 122 or 15.5% of the respondents had a score of either 29 or 30 on the CLS1).

**Table 4:** Descriptive Statistics in the Full Sample (*N* = 768) for the Final CLS Subscales

Variable	Minimum	Maximum	Mean	SD
CLS1 (6 items)	12	30	24.91	3.49
CLS2 (5 items)	5	25	18.01	3.48
CLS3 (8 items)	8	40	29.71	4.42

Correlations, computed from this same full sample, between the CLS subscales and the AQ, SQ, and LSQ are provided in Table 5. Those results indicate that the CLS1 subscale (Need for Clarity/Familiarity) was moderately correlated (i.e., greater than 0.30) with the Attention Switching difficulties (0.314) subscale of the AQ as well as with both the Reflector subscale of the LSQ (0.423; for which high scores indicate a willingness to ponder alternatives and think carefully before taking action) and the Theorist subscale of the LSQ (0.339; for which high scores indicate a willingness to reach answers via logic and to solve problems step-by-step). Certainly, this would then be suggestive of a more deliberate thinking style being employed by individuals scoring higher on the CLS1 subscale. Next, CLS2 (Susceptibility to Load) was correlated at a moderate level with only the Attention Switching difficulties subscale of the AQ (0.483). This is consistent with the fact that this CLS2 factor is measuring attention-related capabilities. Importantly, the partial correlations of both the CLS1 and the CLS2 subscales with Attention Switching difficulties (0.170 and 0.420, respectively, both *p*s < 0.001) were significant indicating that each is related to this AQ facet in a unique fashion. Finally, CLS3 (Conceptual Relations) was moderately correlated with both the Attention to Detail (0.343) and the Imagination difficulties (-0.360) subscales of the AQ and also with the Reflector subscale of the LSQ (0.443). This indicates that all three of these AQ and LSQ facets are subsumed to some extent within the facet captured by the set of CLS3 items. All other correlations in Table 5, although being significant at the 0.05 level if greater than about 0.08, were regarded as not being large enough to try to interpret here (especially given the distinct possibility of some shared method variance). It should be noted, however, that the lack of any moderate-sized correlations between the CLS subscales and the SQ was certainly not expected (although CLS2 and CLS3 were correlated around ± 0.20 with the SQ).

**Table 5:** Correlations in the Full Sample (*N* = 768) Between the Final Three CLS Subscales and all Other Measures

	CLS1	CLS2	CLS3
Social Skills	-.036	.167**	-.179**
Attention Switching	.314**	.483**	-.034
Attention to Detail	.016	-.120**	.343**
Communication	-.155**	.219**	-.258**
Imagination	-.271**	-.001	-.360**
AQ Total Score	-.051	.245**	-.156**
SQ Total Score	-.131**	-.225**	.191**
Reflector	.423**	.122**	.443**
Activist	-.111**	.055	.122**
Pragmatist	.198**	.021	.220**
Theorist	.339**	.064	.260**

^*^ *p* < .05, ** *p* < .01

## Discussion

In the current study, a learning styles scale was developed in an attempt to derive a formal self-report measure of the cognitive-related aspects of learning theorized by Qian and Lipkin [9]. The success of this endeavour was mixed. Whereas it had been anticipated that a factor solution comprised of factors corresponding to LUT- and INT-style learning respectively would emerge, such a solution was not, in fact, obtained. Moreover, some of the items that had specifically been designed to distinguish between LUT and INT learning (e.g., “I tend to remember specific details about things” and “I always try to see connections between things”, respectively) ended up loading together positively on the same factor. As well, a number of items specifically designed to tap into LUT-style learning (e.g., “I like to learn things exactly” and “I like being precise about things”) did not end up coalescing on a unique factor. One reason for this, perhaps, could be that the nature of the processing underlying LUT- and INT-style learning is simply not cognitively accessible. That is, even if an individual tends to learn things in an LUT fashion, he/she may not consciously be aware of such a distinction. Indeed, the fact that the “remember specific details” and “see connections” items mentioned above loaded together suggests that respondents were interpreting these items in terms of an overall ability to grasp and retain conceptual knowledge regardless of its uniqueness or relatedness.

Nonetheless, some indication of the extent to which the current three CLS subscales might indeed be related to the autism spectrum can be discerned from their relations to the AQ subscales. In this regard, both the CLS1 and CLS2 were uniquely related in a positive way to the Attention Switching difficulties subscale of the AQ, with the CLS1 also being related negatively to both Communication and Imagination difficulties and the CLS2 being related negatively to Attention to Detail but positively to both Social Skill and Communication difficulties (albeit at a statistically significant but less than moderate level of correlation). On the other hand, the CLS3 was related positively to the Attention to Detail subscale and negatively to the Imagination difficulties subscale as well as negatively to both the Social Skill and Communication difficulties subscales (albeit at a statistically significant but less than moderate level of correlation). Hence, some degree of convergent validity of the three CLS subscales with a set of scales known to index autistic trait levels was present. Moreover, the fact that the strongest correlations were with the two AQ subscales that could most be regarded as involving cognitive styles (i.e., Attention Switching and Attention to Detail) is indeed supportive of the presence of convergent validity. Importantly, though, it was not the case that any of these correlations were so high as to lead to a consideration of the possibility that any of the CLS scales might simply represent an alternative version of an AQ construct.

Whether the learning styles being measured by the CLS are not being measured by any other scale is a very relevant issue, though. In this vein, an examination by the present authors of the 36 Broader Autism Phenotype Questionnaire [17] items revealed a number of items that could be regarded as being analogous to a CLS-like item “*People have to talk me into trying something new*”, “*I feel a strong need for sameness from day to day*”, “*I am flexible about how things should be done*”, “*I look forward to trying new things*”, “*I like to closely follow a routine while working*”, and “*I keep doing things the way I know even if another way might be better*” which represent 6 of the 12 items of the Rigid subscale (with the other two subscales of this questionnaire being the Aloof and Pragmatic Language subscales). Certainly then, the characteristics being measured by the Rigid subscale of the Broader Autism Phenotype Questionnaire do indeed seem to overlap somewhat with those being measured by the CLS1 (Need for Clarity/Familiarity). An examination of the 6 Repetitive Behavior and 8 Insistence on Sameness items in the 14-item Adult Repetitive Behavior Questionnaire-2 [18], however, did not reveal any items that seemed to be analogous to the ones in the current three CLS subscales. Finally, an examination of the 50 AQ items revealed 4 that seem to be analogous to CLS-like items: “*I usually concentrate more on the whole picture, rather than the small details*” (in Attention to Detail), “*In a social group, I can easily keep track of several different people’s conversations*”, “*I prefer to do things the same way over and over again*”, and “*I find it easy to do more than one thing at once*” (all Attention Switching AQ items).

With respect to the LSQ, moderate-level (or close-to-moderate-level) correlations of both the CLS1 and CLS3 subscales with both the Reflector and Theorist subscales of the LSQ were observed. Interestingly, there does not seem to be any actual item-level overlap between either the CLS1 and CLS3 subscales and the Reflector or Theorist subscales. Hence, it simply seems that those individuals who prefer clarity and familiarity in what they are doing are also more likely to have a preference to think things through carefully and not to jump to conclusions (Reflector qualities) and to be self-disciplined and prefer to deal with people/things in a logical and rational way (Theorist qualities). Similarly, those who feel they can more easily grasp conceptual relations seem to also be those who like to consider all perspectives and carefully weigh alternatives when making decisions (Reflector qualities) as well as to question basic assumptions and think about general principles (Theorist qualities).

More generally, three novel and distinct cognitive learning style subscales emerged from this work. Although, as just discussed, both the CLS1 and CLS3 subscales were indeed correlated with the Reflector and Theorist subscales of the LSQ, each of the CLS subscales could be regarded as representing a specific aspect of cognitive learning style that certainly goes beyond the more general learning style orientations of measures such as the LSI and LSQ. As such, they have the potential to provide more specific insights into the individual learning style differences underlying cognitive processing. It is important to note that, as far as the present authors are aware, no other scales are currently available that tap into individuals’ self-perceptions about how they generally think, learn, and process information. Moreover, inter-individual variability in the performance of cognitive tasks is a ubiquitous phenomena that has received relatively little attention, with such variability almost invariably treated as a nuisance. Hence, the availability of a scale such as the CLS would help a lot with respect to unravelling the underpinnings of such inter-individual variability. Indeed, the present authors plan to use this scale in future work to provide insight into the individual differences that occur within a number of key cognitive paradigms such as those involving numerical processing, categorization, function learning, and decision making. As well, the presence of a scale such as the CLS scale, should then motivate others to develop analogous scales.

Other future work involving the CLS scale should certainly involve attempts to use it (in tandem with the AQ) to predict cognitive performance. As mentioned above, this is indeed something that the present authors are currently in the process of doing. A number of the cognitive tasks that we are currently focussed on studying were specifically chosen to help distinguish between LUT- and INT-style learning (e.g., categorization according to either unidimensional or multidimensional rules). Hence, relating performance on such tasks to measures derived from an explicit consideration of such learning styles could be regarded as providing more information than would be obtained by simply assuming that higher/lower scores on the AQ subscales represent a proxy for LUT/INT learning styles, respectively. Nonetheless, given the nature of the three CLS subscales that emerged, researchers could also use them for more specific purposes such as, say, relating scores on the Susceptibility to Load subscale to performance in a driving simulator.

Regarding their use in such research settings, the three CLS subscales seem quite reliable and user-friendly (in terms of item wordings, directionalities, and the response scale) to use as is. What would not be recommended though is the use of a total 19-item CLS score. That is, the three CLS subscales do indeed seem to be distinct both conceptually and statistically. Note that although the CLS1 is moderately correlated with both the CLS2 and CLS3, those latter two scales are correlated with each other to a much lesser extent. That then implies that CLS1 is related to each of the other two for different reasons. For example, the CLS1 and CLS2 subscales both share variance with the Attention Switching AQ subscale whereas the CLS3 shares variance with the Attention to Detail subscale. On the other hand, whereas both the CLS1 and CLS3 tend to be negatively related to the Communication and Imagination difficulties AQ subscales, the CLS2 is positively related to the Communication and Social Skills difficulties AQ subscales. Hence, the three CLS subscales show qualitatively different patterns of relations with the five AQ subscales.

With respect to the psychometric properties of the CLS scale, attempts to validate its final factorial structure in both community-based and more clinically-based samples would certainly be welcome. Moreover, although the present authors do not have access to samples of individuals on the autistic spectrum, attempts to determine whether scores on the CLS subscales do differ systematically for such individuals would certainly go a long way to connecting both these scales and the underlying learning styles theory upon which they were based to the autism spectrum itself. Finally, it can be noted that the current study took place online. Accordingly, participants did not have a chance to ask questions from researchers while completing the questionnaires. As well, in the online studies, there might be low response rate (16.8% incomplete responses in the present study) and privacy issues [34]. Therefore, it would be of the interest to confirm the three factor-solution of the CLS with a non-online sample.

## Conclusions

The current work aimed to develop a learning style scale in accordance with behavioural and perceptual preferences in autism emphasized in the work of Qian and Lipkin [9]. It was established to address the necessity for a useful and valid tool to measure cognitive learning styles. The result of this work was a novel scale with three subscales that measure distinct facets of how individuals think, learn, and process information. Such a tool can now be used to help understand the nature of the individual differences in cognitive processing that seem to be evident across both the autism spectrum as well as the overall population more generally.

## Data Availability

The datasets used are available from the corresponding author on reasonable request.

## Abbreviations

AC: Abstract Conceptualization
AE: Active Experimentation
AQ: Autism-spectrum Quotient
BAPQ: Broader Autism Phenotype Questionnaire
CE: Concrete Experience
CFA: Confirmatory Factor Analysis
CFI: Comparative Fit Index
CLS: Cognitive Learning Styles
EFA: Exploratory Factor Analysis
INT: Interpolation
LSI: Learning Style Inventory
LSQ: Learning Style Questionnaire
LUT: Look-up-table
RMSEA: Root Mean Square Error of Approximation
RO: Reflective Observation
SPSS: Statistical Package for the Social Sciences
SQ: Systemizing Quotient
SRMR: Standardized Root Mean Square Residual
TLI: Tucker–Lewis Index
